# Mineral Biofortification and Growth Stimulation of Lentil Plants Inoculated with *Trichoderma* Strains and Metabolites

**DOI:** 10.3390/microorganisms10010087

**Published:** 2021-12-31

**Authors:** Roberta Marra, Nadia Lombardi, Alessandro Piccolo, Navid Bazghaleh, Pratibha Prashar, Albert Vandenberg, Sheridan Woo

**Affiliations:** 1 Department of Agricultural Sciences, University of Naples Federico II, 80055 Portici, Italy; nadia.lombardi@unina.it (N.L.); alessandro.piccolo@unina.it (A.P.); 2 Center for Studies on Bioinspired Agro-Environmental Technology (BAT Center), University of Naples Federico II, 80138 Naples, Italy; woo@unina.it; 3 Interdepartmental Research Centre on Nuclear Magnetic Resonance (NMR) for the Environment, Agro-Food and New Materials (CERMANU), University of Naples Federico II, 80055 Portici, Italy; 4 Department of Plant Sciences, University of Saskatchewan, Saskatoon, SK S7N5A8, Canada; navid.bazghaleh@usask.ca (N.B.); pratibha.prashar@usask.ca (P.P.); bert.vandenberg@usask.ca (A.V.); 5 Department of Pharmacy, University of Naples Federico II, 80131 Naples, Italy; 6 National Research Council, Institute for Sustainable Plant Protection, 80055 Portici, Italy

**Keywords:** lentil, biofortification, *Trichoderma*, bioactive metabolites, mineral content, iron, zinc

## Abstract

Biofortification of crops via agricultural interventions represents an excellent way to supply micronutrients in poor rural populations, who highly suffer from these deficiencies. Soil microbes can directly influence plant growth and productivity, e.g., by contrasting plant pathogens or facilitating micronutrient assimilation in harvested crop-food products. Among these microbial communities, *Trichoderma* fungi are well-known examples of plant symbionts widely used in agriculture as biofertilizers or biocontrol agents. In this work, eleven *Trichoderma* strains and/or their bioactive metabolites (BAMs) were applied to lentil plants to evaluate their effects on plant growth and mineral content in greenhouse or field experiments. Our results indicated that, depending upon the different combinations of fungal strain and/or BAM, the mode of treatment (seed and/or watering), as well as the supplementary watering with solutions of iron (Fe) and zinc (Zn), the mineral absorption was differentially affected in treated plants compared with the water controls. In greenhouse conditions, the largest increase in Fe and Zn contents occurred when the compounds were applied to the seeds and the strains (in particular, *T. afroharzianum* T22, *T. harzianum* TH1, and *T. virens* GV41) to the soil. In field experiments, Fe and Zn contents increased in plants treated with *T. asperellum* strain KV906 or the hydrophobin HYTLO1 compared with controls. Both selected fungal strains and BAMs applications improved seed germination and crop yield. This biotechnology may represent an important challenge for natural biofortification of crops, thus reducing the risk of nutrient deficiencies.

## 1. Introduction

The FAO estimates that by 2050 the world population will reach 9.1 billion people and food production should increase by 70% [1]. However, the availability of agricultural land is reducing more and more, and many of the natural resources currently used by humans show signs of degradation or pollution. Moreover, basic crops (i.e., rice, wheat, corn), the main food in diets of the poorest populations, contain limited amounts of micronutrients, including minerals (iron, zinc, iodine, selenium, etc.) and vitamins, thus not guaranteeing the recommended daily requirements [2,3].

The spread of micronutrient malnutrition (MNM), a chronic shortage of minerals and vitamins, has serious socio-economic consequences, both individually and collectively [3,4]. This “hidden hunger” is known to induce diseases and disorders in both developed and developing nations and is currently considered as one of the most serious challenges that faces humanity [3,5]. Indeed, interventions to reduce micronutrient deficiencies were listed in the Copenhagen consensus as the number one priority, in agreement with the Sustainable Development Goal 2 (SDG2) established by the United Nations in 2015 [6].

MNM can be effectively countered in different ways, e.g., by encouraging dietary diversification, by supplementing micronutrients in the form of pills or enriched processed foods or through biofortification, intended as the increase in bioavailable micronutrients in the edible parts of staple crops. The latter comprises conventional plant breeding techniques, agronomic approaches, as well as genetic engineering methods [2,7]. Several studies have shown the positive effects of consuming biofortified crops to contrast hidden hunger also in the long-term [8]. However, each strategy has advantages and disadvantages, and the results are often limited by infrastructures, regulations, and costs [9,10].

Agronomic biofortification may offer novel opportunities to both increase the micronutrients content in crops and reduce the use of chemical fertilizers, which represent a cost to farmers and pose a risk to the human health and environment. A novel method to increase the bioavailability of important micronutrients to the plant consists of the inoculation with beneficial microorganisms that can favor the solubilization, absorption, and assimilation of these compounds by the plant [11,12]. These include endophytic fungi and yeasts, that enhance plant growth directly by improving plant nutrition and are largely applied as biofertilizers worldwide [13,14]. Moreover, indirect beneficial effects of endophytic colonization include resistance induction, tolerance towards biotic and abiotic stresses, production of antimicrobial and useful compounds, etc. [14]. Among plant growth-promoting microbes (PGPM), several species of the genus *Trichoderma* have proven ability to establish symbiotic relationships with host plants [15]. *Trichoderma* are soil fungi recognized as beneficial biological control agents against plant pathogens for more than 70 years. At present, they are recognized as active ingredients in more than 250 agricultural products marketed worldwide as biological pesticides, fertilizers, plant growth enhancers and stimulants of natural resistance, as well as soil amendments/inoculants [16]. An increasing number of studies are reporting the effectiveness of new species or isolates of *Trichoderma* to improve plant fitness under abiotic stresses [17], activate plant defense responses [18], stimulate crop productivity and nutrient content [19,20,21] or control plant pathogens [15]. These effects have been also related to the production of *Trichoderma* bioactive metabolites (BAMs), that can be used either alone or in combination with the living microbe [22].

The aim of this work was to use a technology based on microbial fungal organisms and/or their extracts to augment the nutritional value, specifically to increase the mineral content, in an important legume crop, such as lentil (*Lens culinaris* L.). Legumes are key sources of dietary proteins and their consumption has numerous positive effects on health, including reduced risk of several chronic diseases, improvement of the digestive function, and prevention of obesity [23]. Therefore, we tested the effects of biocontrol agents, such as fungi of the genus *Trichoderma*, and/or their bioactive metabolites (BAMs), on the uptake capacity of important minerals in a lentil variety that is most widely cultivated throughout the world. The bioformulations were applied under different conditions and in diverse combinations in order to identify and select a treatment that may be effectively applied to the crop in question for mineral biofortification. Furthermore, the effects of the different treatments in terms of promotion of plant growth and yield increase were evaluated.

## 2. Materials and Methods

### 2.1. Plant Material

Lentil (*Lens culinaris* L.) variety CDC MAXIM CL was used for the experiments. The characteristics of this variety of red lentil, which is among the most cultivated varieties in the world, are summarized in Table 1.

### 2.2. Fungal Strains and Metabolites

Pure cultures of 11 *Trichoderma* strains, including *T. harzianum* strain TH1 (TH1), *T. harzianum* strain M10 (M10), *T. longibrachiatum* strain MK1 (MK1), *T. atroviride* strain P1 (P1), *T. afroharzianum* strain T22 (T22; formerly *T. harzianum*), *T. asperellum* strain T53 (T53), *T. harzianum* strain HK2 (HK2), *T. harzianum* strain HK5 (HK5), *T. asperellum* strain CRN1 (CRN1), *T. asperellum* strain KV906 (KV906), and *T. virens* strain GV41 (GV41) were obtained from the collection available at the Department of Agricultural Sciences of the University of Naples Federico II, Italy (Table 2). Fungal cultures were cultivated on Petri plates at 25 °C containing Potato Dextrose Agar (PDA, HiMedia Laboratories, Mumbai, India). After 7–10 d, the spores were collected, and the concentration of conidial suspensions was determined using a counting chamber. Fungal cultures were identified by microbiological and molecular techniques. The strains used in this work were selected according to their biocontrol and/or plant growth promotion activities as determined in previous experiments carried out in our laboratory.

In this work, three different *Trichoderma* metabolites were used (Table 3): (i) 6-Pentyl-alpha-pyrone (6PP) isolated from *T. atroviride* strain P1 (yield 84 mg/L of culture filtrate), which has been previously described for its activity and role in plant growth promotion [25]. (ii) Harzianic acid (HA) produced by *T. harzianum* strain M10 (yield 98 mg/L of culture filtrate), which is a tetramic acid with proven capability to bind iron, stimulate plant growth, and exert antifungal activity [26,27]. Previous results indicated that this metabolite acts as a siderophore causing iron precipitation [28]. (iii) The hydrophobin HYTLO1, a small protein (8 kDa) secreted by *T. longibrachiatum* strain MK1. This protein is involved in the formation of aerial mycelium, has antifungal activity, and is able to stimulate plant defense to pathogen attack, as well as root growth [28]. These compounds were extracted and characterized as previously described [25,26,27,28]. BAM solutions were prepared by diluting the compound with distilled water to the final concentration used for the treatments [21]. For HA and 6PP, 0.1% ethyl acetate was added to facilitate resuspension and successively evaporated under cabinet flow.

### 2.3. Experimental Design

For each experiment, lentil seeds were previously sterilized by immersion in 1% (*v*/*v*) sodium hypochlorite solution for 5 min, followed by repeated washes in sterile distilled water. Then, the seeds were immersed in a solution of ethanol (70%, *v*/*v*) for 5 min to promote germination.

Fungal strains or BAMs were applied to: (i) The seeds by dipping in *Trichoderma* spore suspensions or metabolite solutions. Seeds were left in Petri plates under laminar flow hood until the solutions were fully absorbed. The controls consisted of water-treated seeds. (ii) The soil by watering in proximity of the root crown. Seven days after the treatment, 50% of the plants were watered with a solution containing Fe and Zn (1X FeZn solution), consisting of 27.8 mg/L iron sulfate heptahydrate and 8.6 mg/L zinc sulfate heptahydrate, respectively. Higher mineral contents (2X, 5X, 10X) were obtained by proportionally increasing the Fe and Zn concentrations. The remaining plants were watered only with water. Detailed protocols used in each experiment are reported below.

#### 2.3.1. EXPERIMENT #A: Application of *Trichoderma* Strains or BAMs in Greenhouse

The seeds were treated with a spore suspension (1 × 10^7^ sp/mL) of different *Trichoderma* strains (M10, P1 or T22) or with a solution of the secondary metabolites HA or 6PP (both at 10^−6^ M). The seeds were placed to germinate in pots of 14 cm diameter containing sterile soil (10 seeds per pot with three replicates for each treatment) and after 3 d the percentage of germination was evaluated. The pots were setup in a completely randomized design, and the plants were grown under greenhouse conditions (day/night temperature 25/15 °C; humidity 50−60%; day length 12–14 h) and watered when necessary. About 4 weeks after sowing, a first soil watering with 25 mL/pot of the same solutions containing the spores or the BAMs used for seed treatments was carried out. After 7 d, 50% of the plants were treated with 25 mL/pot of 1X FeZn solution and the remaining plants with water (Table S1). Plants were harvested 2 weeks later and the pods containing lentil seeds were collected for further analyses.

#### 2.3.2. EXPERIMENT #B: Application of *Trichoderma* Strains in Greenhouse

In this experiment, 11 strains of *Trichoderma*, previously selected and characterized (par. 2.2), were used. The treatments consisted of two applications of spore suspensions (1 × 10^7^ sp/mL) dissolved in water, first to the seeds and then, 4 weeks later, to the soil. Water treatments served as controls. After 7 d, another soil watering was carried out using 1X FeZn solution, prepared as described above. The experimental conditions (number of seeds per pot, replicates per treatment, etc.) were the same as in experiment #A. Plants were harvested 2 weeks later and the pods containing lentil seeds were collected for further analyses.

#### 2.3.3. EXPERIMENT #C: *Trichoderma* Strains and/or BAMs Applied Singly or Combined in Greenhouse

In this experiment, *Trichoderma* strains (T22, TH1, GV41) and metabolites (HA, 6PP, HYTLO1) were applied singly to the seeds (Table S2). The metabolites HA and 6PP were both used at a final concentration of 10^−6^ M, while HYTLO1 was used at 10^−8^ M. Water-coated seeds served as controls. The seeds were placed in 14 cm diameter pots filled with sterile soil. In each pot, seven seeds were sowed, with three replicates per treatment. The pots were setup in a completely randomized design, and the plants were grown under greenhouse conditions (day/night temperature 25/15 °C; humidity 50−60%; day length 12–14 h) and watered when necessary. The first soil watering was made 2 months after sowing, using 35 mL of solution per pot. After 7 d, 50% of the plants were treated with 35 mL of 1X FeZn solution and the remaining plants with water (Table S2).

#### 2.3.4. EXPERIMENT #D: Plant Treatments in Field Conditions

A field experiment was performed in a trial at the Department of Agricultural Sciences of the University of Naples Federico II located in Portici (Naples, Italy). The plot size was approximately 15 × 7 m. The soil of the site was clay loam with pH (1:2.5 soil:water) 4.8, non-saline [EC(1:2.5 soil:water) 0.3 dS/m], and with cation exchange capacity of 13.57 cmol (p+)/kg. The experimental soil contained 1.5% organic carbon, available nitrogen (N; 296 kg/ha), available phosphorus (P; 27 kg/ha), and available potassium (K; 278.16 kg/ha). The climate of the region is Mediterranean. The experimental site is located at 40.81° N latitude, 14.34° E longitude, and 47 m above the mean sea level. A commercial nodulating bioformulate based on selected nitrogen fixing rhizobia (Umostart, Sipcam, Italy) was applied according to the manufacturer’s instructions. The formulation consists of a consortium of *Bradyrhizobium japonicum*, *Rhizobium melioti*, and *Rhizobium leguminosarum* bv. *viciae*, in addition to a nutritional substrate that promotes root development at an early stage.

Lentil seeds were coated with 500 µL of *Trichoderma* spore suspensions (from strains TH1, M10, P1, T22, T53, HK5, CRN1, KV906 or GV41) or metabolite solutions (HA, 6PP or HYTLO1). *Trichoderma* spore suspensions were used at 1 × 10^7^ spore/mL. The metabolites HA and 6PP were both used at a final concentration of 10^−6^ M, while HYTLO1 was used at 10^−8^ M. The seeds were germinated in polystyrene boxes. Each treatment consisted of 10 plants, with three biological replicates. After 10 d, the seedlings were transplanted into soil, with 15 cm plant intervals, while 50 cm distance was used to avoid contaminations among the treatments. For the combined application [T22 + HA], the metabolite was added to the spore suspension at the time of inoculation. The crop was harvested at physiological maturity after 4 months.

### 2.4. Plant Analysis

Plant samples consisted of lentil pods containing the seeds. The growth and yield parameter measured included: Seed germination (%), plant dry weight (g/plant), and seed weight (g/plant).

Determination of Mineral Content

In this work, the analysis of the mineral content (Fe, Zn) of lentil samples was carried out on mineralized samples, as described by [29]. The samples were dried in an oven (60 °C) for about 3 days (until complete dehydration), and subsequently were finely ground in a pulverizer, using a cycle of 5 min at a speed of 550 rpm. For each sample, 500 mg of powder were transferred into porcelain crucibles and incinerated in a muffle furnace at 500 °C for 4 h. The resulting ash, once cooled, was dissolved in 5 mL of nitric acid (HNO_3_, diluted 1:3, *v*/*v*) and boiled until complete evaporation of the acid solution. The samples were placed again in muffle at 500 °C for 1 h and then left to cool. Then, 5 mL of hydrochloric acid (HCl, diluted 1:3, *v*/*v*) and 15 mL of MilliQ grade water were added and the solution was boiled for 2 min and filtered (Macherey-Nagel MN640d filters) to remove any corpuscular particles that may interfere with the analysis. The filtered solution was collected in 25 mL flasks and brought to volume with MilliQ water. The resulting solutions were stored at 4 °C until analyzed.

The mineral content of the samples was determined by Atomic Absorption Spectroscopy, using the device AAnalyst 700 (Perkin-Elmer, Waltham, MA, USA). For each item analyzed, appropriate calibration curves were constructed using commercial standard solutions (Figure S1). All of the measurements were carried out using the operating flame standard conditions recommended by the manufacturer. Each measurement is the average of three consecutive readings taken by the instrument in succession. Three biological replicates were analyzed for each sample. For all of the samples, the glassware was washed with an aqueous solution of 2% (*v*/*v*) HNO_3_.

### 2.5. Statistical Analysis

All of the obtained data were analyzed by one-way ANOVA using SPSS 15.0 software (SPSS, Inc., Chicago, Illinois), and significant differences among the treatments were separated using S−N−K (Student− Newman−Keuls) and Fisher’s Least Significant Difference (LSD) post hoc tests at the 0.05 level of significance.

## 3. Results

### 3.1. Effects of Trichoderma Strains or BAMs on Lentil Plants in Greenhouse (#Experiment A)

Our results indicated that the application of *Trichoderma* strains or metabolites to lentil plants affected the assimilation of minerals in the plant. Depending upon the different combinations of fungal strain and/or BAM, the mode of treatment (seed and/or watering), as well as the supplementary watering with FeZn solution, the mineral absorption in the treated plants varied as compared with the water controls. Preliminary experiments showed that the biocontrol agent *T. afroharzianum* strain T22 applied as a seed-coating produced an increase in iron and zinc contents of lentil plants watered with a FeZn solution (data not shown). Higher concentrations (2X, 5X or 10X) of FeZn solution did not ensure a proportional accumulation of these minerals in the treated samples. In addition, the highest concentration (10X) showed phytotoxic effects on plants when applied in combination with the *Trichoderma* strain. Therefore, the 1X FeZn solution was used in subsequent experiments.

**Figure 1:**
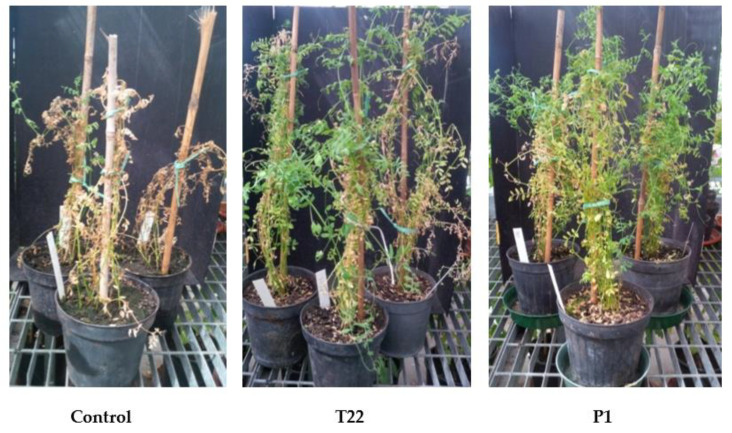
Development of lentil plants treated with various *Trichoderma* strains. (**Left**) Control (water) untreated. (**Center**) Plants treated with *T. afroharzianum* strain T22. (**Right**) Plants treated with the strain P1 of *T. atroviride*.

**Figure 2:**
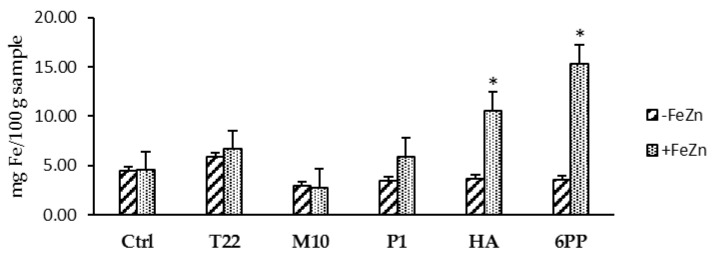
Iron content in lentil plants treated with *Trichoderma* strains (T22, M10 or P1) or metabolites (HA or 6PP) to the seeds and to the soil (1st watering). Water treatments served as controls (Ctrl). Data represent the mean value of three biological replicates ± standard error. The second soil watering was performed with a 1X solution of iron and zinc (+FeZn) or water (−FeZn). Asterisks indicate significant differences (*p* < 0.05) compared with the control. Statistical analysis was carried out using the one-way ANOVA.

Seed treatments followed by soil watering with strain or metabolite solutions determined an increase in plant development when lentil plants were grown in greenhouse. The growth and physiology of the treated plants were enhanced, with a greater ability to flower and produce fruits. The treated plants were greener and demonstrated a prolonged development compared with the untreated controls, which appeared to have an advanced state of senescence (Figure 1). In addition, the metabolites HA and 6PP determined the highest increase in iron content in treated plants compared with controls when plants were watered with the FeZn solution (Figure 2). No effect was observed in zinc content in the treated plants compared with controls.

### 3.2. Effects of Trichoderma Strains on Lentil Plants in Greenhouse (#Experiment B)

**Figure 3:**
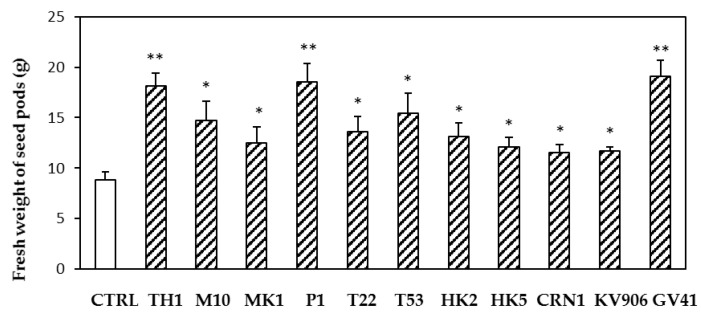
Effects of treatments based on different *Trichoderma* strains (TH1, M10, MK1, P1, T22, T53, HK2, HK5, CRN1, KV906, GV41) on the yield of lentil plants (expressed as fresh weight of seed pods per treatment). Data represent the mean value of three biological replicates ± standard error. The treatments consisted of two applications of spore suspensions, first to the seeds and then, 4 weeks later, to the soil. Water treatments served as controls (CTRL). After 7 d, another soil watering was carried out using the 1X FeZn solution. Asterisks indicate significant differences (* *p* < 0.05; ** *p* < 0.01) compared with the control. Statistical analysis was carried out using the one-way ANOVA.

Applications of different *Trichoderma* strains in greenhouse affected both crop productivity and mineral content. Compared with the untreated control, all of the strains determined a significant increase (*p* < 0.05) in fresh weight of the harvested pods (Figure 3). Highest increases (*p* < 0.01) in crop yield were observed after the treatments with strains TH1, P1, and GV41. Moreover, several strains were able to increase the content of iron (T22, TH1, and GV41) and zinc (T22) in the treated lentils (Table 4).

### 3.3. Effects of Trichoderma Strains or BAMs Applied Singly or Combined (#Experiment C)

**Figure 4:**
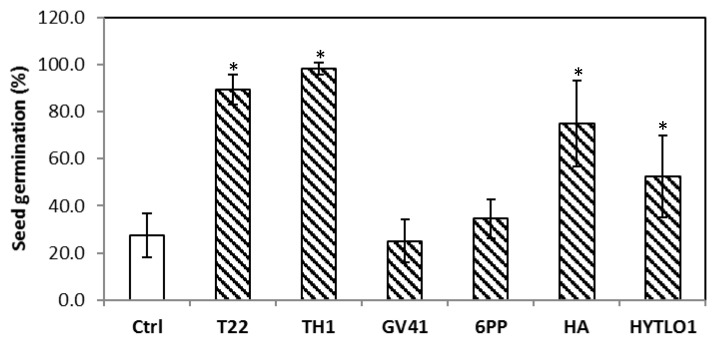
Effect of the seed treatment with *Trichoderma* strains (T22, TH1, GV41) or metabolites (6PP, HA, HYTLO1) on the germination of lentil seeds cv. CDC MAXIM CL (3 days after the treatment). Water treatments served as controls (Ctrl). Asterisks indicate significant differences (*p* < 0.05) compared with the control. Statistical analysis was carried out using the one-way ANOVA.

Based on these data, the strains that showed the best performances were applied singly or combined in greenhouse (exp. #C, par. 2.3.3). The seed treatment with *Trichoderma* spores or BAMs differently affected germination at 3 days after sowing (Figure 4). *Trichoderma* strains T22 and TH1 significantly (*p* < 0.05) increased about 70% of germinated seeds as compared with water controls. Similarly, the seeds coated with the metabolite HA or HYTLO1 solutions showed, respectively, about 60 and 50% higher germination vs. control (Figure 4).

When singly or combined applications of *Trichoderma* strains and BAMs were carried out in greenhouse, significant increases in mineral content occurred, particularly when plants were supplemented with a FeZn solution. In single treatments (strains/metabolites and water or vice versa), the highest increases in iron content were observed when the strain TH1 was applied to the seed (treatment TH1/H_2_O; Table 5) or when the BAMs 6PP or HYTLO1 were watered to the soil (treatments H_2_O/6PP and H_2_O/HYTLO1, respectively; Table 5). In the absence of supplementation with the FeZn solution (−FeZn), significant (*p* < 0.05) increases in iron content were observed only when strains T22 or GV41 were applied as soil watering (treatments H_2_O/T22 and H_2_O/GV41, respectively; Table 5). In the case of zinc content, treatments H_2_O/T22 and H_2_O/HA gave significant differences compared with water controls (in the absence of FeZn supplementation). Unfortunately, after germination, all of the T22/H_2_O-treated plants supplemented with FeZn solution died. Therefore, their mineral content was not determined (Table 5).

In the same experiment, the effects of combined applications of *Trichoderma* BAMs to the seeds/spores and to the soil on the lentil mineral content were evaluated (Table 6). The iron content increased significantly (*p* < 0.05) in plants whose seeds were treated with HA, 6PP or HYTLO1, and then watered, respectively, with water, T22 or G41 and TH1, in the absence of FeZn supplementation (treatments HA/H_2_O, 6PP/H_2_O, 6PP/T22, 6PP/GV41, HYTLO1/TH1; Table 6). Conversely, the iron content quadruplicated when the combined treatments HA/GV41 and 6PP/GV41 were used, followed by 1X FeZn watering (Table 6). Only the seeds coated with HA or HYTLO1 that were successively watered with water and GV41 spores, respectively, in addition to the 1X FeZn solution, revealed a significant increase in zinc content compared with controls (Table 6). Overall, the supplementation with 1X FeZn solution increased the content of iron in treated plants, while an opposite trend was found in the case of zinc (Table 6).

### 3.4. Effects of Trichoderma Strains and/or BAMs on Lentil Plants in Field Conditions (#Experiment D)

A significant promotion of plant growth and yield increase was observed also in field conditions when lentil seeds were coated with *Trichoderma* strains and/or BAMs (Table 7). The plant dry weight vs. control increased by about 120 and 57% when strains M10 or TH1, respectively, were used. Interestingly, the production of seed weight augmented up to 169% in TH1-treated plants compared with control (Table 7). Other treatments showing significant increases of seed weight/plant were those performed with strains M10 (+112% vs. CTRL), T53 (+71%), HK5 (+53%), KV906 (+81%), GV41 (+65), and with the metabolites 6PP (+36%) or HYTLO1 (+147%). Regarding the mineral content, seed coating with strain KV906 or the BAM HYTLO1 determined the highest increases in both iron and zinc contents compared with water controls (Table 7). However, no significant differences in terms of promotion of plant growth, productivity or in terms of an increase in the mineral content were observed following the combined application of strain T22 and the metabolite HA when lentil plants were grown in field trials.

## 4. Discussion

Lentils are cultivated worldwide, especially in non-optimal, temperate agricultural zones. The major global producers in 2019 were Canada and India, accounting for 37 and 21%, respectively, of the global production [30]. Lentils are an excellent source of micronutrients, particularly iron (Fe) and zinc (Zn). In addition, they are low in fat, contain a high amount of protein (average 23%), and are rich in some essential amino acids (such as lysine and phenylalanine) [31]. The biofortification of crops with microorganisms represents an innovative strategy to increase the nutritional content of crops, thus contributing to contrast the malnutrition [23,32,33]. In this work, the use of a microbial technology, based on antagonistic fungi of the genus *Trichoderma* and/or their metabolic products has been proposed in order to increase the nutritional value of an important legume, such as lentil. *Trichoderma* spp. are widely used in agriculture as biocontrol agents and are well known for their ability to establish complex interactions with plants, promoting their growth, inducing systemic resistance, and increasing the tolerance to abiotic stresses [15,16,34]. Recently, Bazghaleh et al. showed that the lentil genotype greatly influenced the colonization of root-associated fungi, including *Trichoderma* beneficial species, and affected the biocontrol against the oomycete *Aphanomyces euteiches* [35].

We tested several species and strains of *Trichoderma*, which include isolates that are able to exert beneficial effects on plants. Eleven strains, identified by morphological characters and molecular techniques, have been used in this study. Moreover, the experimental design included three BAMs (6PP, HA, and HYTLO1) produced by *Trichoderma* strains, previously isolated and characterized and known for their ability to control pathogens and establish beneficial interactions with plants [25,26,27,28]. Among these metabolites, HA showed the ability to chelate iron [27]. The iron sequestration can regulate the competition for nutrients that develop between soil microorganisms [36] and may represent one of the mechanisms of active biocontrol against plant pathogens [15,37,38].

Here, in greenhouse and field assays, we have performed an evaluation of the effects of strains or metabolites of *Trichoderma* on the growth and mineral content of lentil plants of the Canadian variety CDC MAXIM CL. The analyses focused mainly on iron and zinc, since these minerals are among the micronutrients that are most frequently deficient in human nutrition, but present in legume pulses [23,39,40]. The seed treatment with *Trichoderma* strains or metabolites affected the germination of lentil seeds, according to the strain/compound used in greenhouse experiments (Figure 4). Up to an approximately 70% increase in seed germination was found in TH1- and T22-treated samples compared with the untreated control. In addition, significant improvements were obtained with the BAMs HA and HYTLO1. When seed coating was followed by soil washing with *Trichoderma* spore suspensions/BAMs solutions and mineral fertilization, plants showed a better appearance compared with water-controls, in terms of development, physiology, and productivity (Figure 1 and Figure 3). In field conditions, the highest improvements of plant dry weight and seed weight were observed following seed coating with M10 and TH1 *T. harzianum* spores (Table 7). Similar biostimulant activities exerted by *Trichoderma* species and their metabolites have been well documented on different crops [17,19,20,41,42,43], including legumes [21,34,44,45]. Experimental evidence indicated that the use of plant growth-promoting microbes and their bioactive compounds may represent a valid strategy to withstand biotic and abiotic stresses that could affect plant yield and performance [17,46,47,48,49].

In our study, the mineral content of lentil plants was affected differently by the seed/soil treatment with *Trichoderma* spores/BAMs, as well as by the mode and number of applications. Significant (*p* < 0.05) increases in Fe were observed in plants treated with strains T22, TH1, and GV41 (Table 4) or with the compounds HA and 6PP (Figure 2). Similarly, the content of Zn was affected by *Trichoderma* applications (Table 4). Some studies showed that several species of *Trichoderma* (particularly *T. asperellum*) can increase the concentration of iron and other minerals in wheat and lupine plants [11,12]. Previously, Altomare et al. have demonstrated the ability of T22 strain of *T. harzianum* to solubilize poorly soluble phosphates and mineral compounds (including Fe_2_O_3_, MnO_2_, Zn) through chelation and reduction mechanisms, which are also involved in biocontrol processes against plant pathogens and, probably, in promoting the plant growth [50].

Furthermore, we tested if combinations of the living microbe and bioactive molecules in repeated applications (to the seed and then to the soil) could exert a better effect than each component applied singly. Our data indicated that under greenhouse conditions a combined application of spores and BAMs or each component applied singly increased iron content (Table 5 and Table 6). Overall, single treatments consisting of soil watering with *Trichoderma* strains or BAMs determined the higher increase in Fe content compared with seed coating, both in the presence or absence of mineral fertilization (±1X FeZn solution). However, the highest Fe contents were found in plants treated with the combination of HA or 6PP applied to the seeds and the strain GV41 watered to the soil (Table 6). In addition, the field experiment revealed a significant increase in Fe and Zn contents in lentil plants treated with strain KV906 or the hydrophobin HYTLO1, both used as seed treatments (Table 7). This finding underlines the importance of correct formulation and timing for the application of bioformulates consisting of living microbes and/or natural compounds.

The results obtained in this work indicate that the application of selected strains of *Trichoderma* to lentil plant may improve the nutritional value of these food crops, i.e., by increasing the contents of important mineral micronutrients, such as iron and zinc. In a previous study, we demonstrated that the application of selected *Trichoderma* strains and metabolites enhanced the productivity of soybean and improved the lipid content in this oilseed crop [21]. Similarly, Yadav et al. investigated the effects of microbial applications based on *Pseudomonas* and *Trichoderma* inocula on the growth and nutritional value of chickpea [51]. A fusant strain of *Trichoderma* showed the ability to increase amino acids and the mineral content of chickpea [44]. However, the effects on plant growth and productivity, as well as on mineral content, varied not only according to the strain/compound used, but also to the mode of application (seed/soil treatments) and plant growth conditions (greenhouse/field). This finding underlines the importance in defining opportune selection processes to optimize the outcomes resulting from biofortification or plant growth promotion strategies.

The use of BAMs produced by these fungi can represent a valid alternative to the use of the living microorganism, offering a comparable effectiveness and, in some cases, superior to the producer strain. The production of siderophores by *T. asperellum*, for example, reduced the effects of salt stress in cucumber plants thanks to the ability of these extracts to chelate or solubilize the iron by reducing Fe^3+^ to Fe^2+^ [52].

The proposed microbial biotechnology could broaden the concept of traditional agronomic biofortification, usually consisting of the administration of fertilizers containing essential mineral micronutrients. This strategy intends to promote the use of organisms and/or natural substances to obtain numerous benefits, ranging from pest control to the promotion of plant growth, induction of resistance to improve the quality of crops, with obvious positive implications on human and animal nutrition. To this, the economic benefits and the opportunity to build a new biotechnology can be added. This increases the nutritional value of the crop with deployment times that are below those required for the application of genetically improved crops [53].

## 5. Conclusions

The agronomic biofortification using beneficial microbes or their metabolites intends to contribute to the prevention of MNM. Our results confirmed that *Trichoderma* strains and their BAMs affect the growth, productivity, and mineral content of lentil plants. Here, the highest increase in plant growth and productivity was obtained using *Trichoderma* strain TH1, both in greenhouse and field conditions. In addition, combined applications of BAMs to the seeds and strains (in particular, T22, TH1 or GV41) to the soil determined the higher increases in mineral content compared with the control, regardless of additional fertilization. However, the type and mode of application, as well as the plant growth conditions determined differences in treated plants. Our findings underline the importance of properly selecting the best strain/compound and its formulation to achieve the highest performance, according to the desired task. Additional extensive studies are needed to evaluate whether the increase in the minerals obtained with the use of bioformulates corresponds to a greater bioavailability of micronutrients for the final consumer of the food. Similarly, it is necessary to clarify whether the effects observed in this study can be played on other varieties of lentils or other legumes.

## Figures and Tables

**Table 1:** Characteristics of lentil (*Lens culinaris* L.) variety CDC MAXIM CL used in this study [24].

Variety	CDC MAXIM CL
**Seed color**	Red
**Seed dimension**	Small
**Maximun yield (%)**	100
**Height (cm)**	34
**Flowering (days** **)**	51
**Seed weight (g/1000)**	40
**Aschochyta Resistance**	Good
**Anthracnose resistance (Race 1)**	Good

**Table 2:** List of *Trichoderma* species and strains used for the experiments.

No.	*Trichoderma* Species	Strain	Origin
1	*harzianum*	TH1	Italy
2	*harzianum*	M10	Australia
3	*longibrachiatum*	MK1	Italy
4	*atroviride*	P1	Norway
5	*afroharzianum*	T22	USA
6	*asperellum*	T53	Spain
7	*harzianum*	HK2	USA
8	*harzianum*	HK5	USA
9	*asperellum*	CRN1	Costa Rica
10	*asperellum*	KV906	Brazil
11	*virens*	GV41	USA

**Table 3:** *Trichoderma* bioactive metabolites (BAMs) used in this work.

Metabolite	Chemical Feature	Source	Properties	Reference
6-pentyl-alpha-pyrone (6PP)	Pyrone	*T. atroviride* strain P1	Antibiotic, volatile compound with a characteristic smell of coconut.	[25]
Harzianic acid (HA)	Tetramic acid	*T. harzianum* strain M10	Antifungal activity, promotion of plant growth, ability to bind iron.	[26,27]
HYTLO1	Hydrophobin	*T. longibrachiatum* strain MK1	Involved in the aerial mycelium formation and stimulation of plant defenses and root growth, antifungal activity.	[28]

**Table 4:** Effects of *Trichoderma* strains (TH1, M10, MK1, P1, T22, T53, HK2, CRN1, KV906, GV41) on the lentil mineral content. Water treatments served as controls (CTRL). Data represent the mean value of three biological replicates ± standard deviation (SD). Different letters in a column indicate statistically significant differences for *p* < 0.05.

	Mineral Content (mg/100 g Sample)	
Strain	Fe	Zn
TH1	5.82 ± 0.65 bd	7.345 ± 0.84 a
M10	4.02 ± 0.41 a	7.245 ± 0.80 a
MK1	2.77 ± 0.28 c	7.695 ± 0.69 a
P1	4.75 ± 0.39 ad	6.845 ± 0.77 c
T22	6.47 ± 0.45 b	9.395 ± 0.85 b
T53	3.99 ± 0.52 a	7.295 ± 0.64 a
HK2	4.42 ± 0.68 a	7.345 ± 0.25 a
HK5	4.01 ± 0.54 a	7.245 ± 0.84 a
CRN1	3.15 ± 0.27 c	7.745 ± 0.67 a
KV906	4.22 ± 0.51 a	7.545 ± 0.64 a
GV41	6.88 ± 0.52 b	7.695 ± 0.34 a
Control	4.00 ± 0.63 a	7.995 ± 0.40 a

**Table 5:** Effects of *Trichoderma* strains (T22, TH1, GV41) or metabolites (HA, 6PP, HYTLO1), applied singly, on the lentil mineral content. Water treatments served as controls (CTRL). Treatments were applied to the seed (indicated on the left), then to the soil (indicated on the right). After 7 d from the soil treatment, another soil watering was carried out using the 1X FeZn solution (+FeZn) or water (−FeZn). Data represent the mean value of three biological replicates ± standard deviation (SD). Different letters in a column indicate statistically significant differences for *p* < 0.05; nd: Not determined.

	Mineral Content (mg/100 g Sample)							
Treatment Applied to Seed/Soil	IRON CONTENT				ZINC CONTENT			
	−FeZn		+FeZn		−FeZn		+FeZn	
CTRL	5.60	±0.41 a	12.06	±0.84 a	6.00	±0.62 a	4.28	±0.45 a
H_2_O/T22	13.59	±1.63 b	4.57	±0.35 b	7.95	±0.84 b	3.81	±0.25 a
H_2_O/TH1	4.21	±0.58 c	4.55	±0.65 b	6.31	±0.36 a	4.62	±0.41 a
H_2_O/GV41	13.88	±1.28 b	10.77	±0.96 a	4.85	±0.58 a	4.46	±0.56 a
H_2_O/HA	7.13	±0.63 d	7.21	±0.78 c	7.52	±0.89 ab	4.28	±0.69 a
H_2_O/6PP	4.38	±0.48 c	24.99	±1.96 d	6.40	±0.54 a	4.47	±0.85 a
H_2_O/HYTLO1	3.36	±0.36 c	47.89	±1.84 e	5.82	±0.23 a	3.79	±0.55 a
T22/H_2_O	5.56	±0.69 ac	nd		4.90	±0.85 a	nd	
TH1/H_2_O	7.73	±0.36 d	19.53	±2.65 d	5.53	±0.96 a	5.29	±0.98 a
GV41/H_2_O	5.73	±0.44 a	2.81	±0.24 f	6.08	±0.65 a	4.04	±0.63 a
HA/H_2_O	7.76	±0.18 d	5.51	±0.75 c	5.69	±0.41 a	5.13	±0.35 a
6PP/H_2_O	9.13	±0.85 e	8.57	±0.96 cg	6.93	±0.21 a	4.56	±0.14 a
HYTLO1/H_2_O	7.02	±1.00 ad	9.94	±1.45 ag	6.61	±0.36 a	5.01	±0.56 a

**Table 6:** Effects of combined treatments with *Trichoderma* strains (T22, TH1, GV41) or metabolites (HA, 6PP, HYTLO1) on the lentil mineral content. Water treatments served as controls (CTRL). Treatments were applied to the seed (indicated on the left), then to the soil (indicated on the right). After 7 d from the soil treatment, another soil watering was carried out using the 1X FeZn solution (+FeZn) or water (−FeZn). Data represent the mean value of three biological replicates ± standard deviation (SD). Different letters in a column indicate statistically significant differences for *p* < 0.05.

	Mineral Content (mg/100 g Sample)							
Treatment Applied to Seed/Soil	IRON CONTENT				ZINC CONTENT			
	−FeZn		−FeZn		−FeZn		+FeZn	
CTRL	5.60	±0.41 a	12.06	±2.56 a	6.00	±0.62 a	4.28	±0.44 a
HA/H_2_O	7.76	±0.18 bc	5.51	±0.75 b	5.69	±0.41 ab	5.13	±0.35 b
HA/T22	6.31	±0.56 a	12.46	±1.69 a	5.23	±0.63 ab	4.00	±0.72 a
HA/TH1	6.26	±0.98 a	13.21	±1.45 a	5.28	±0.66 ab	3.61	±0.35 a
HA/GV41	6.29	±0.68 a	46.74	±6.51 c	4.39	±0.58 b	4.37	±0.68 a
6PP/H_2_O	9.13	±0.85 c	8.57	±0.96 b	6.93	±0.21 a	4.56	±0.14 a
6PP/T22	7.97	±0.77 bc	13.29	±1.69 a	5.36	±0.81 ab	4.61	±0.35 a
6PP/TH1	3.91	±0.45 d	4.41	±0.87 b	5.08	±0.80 ab	4.49	±0.16 a
6PP/GV41	7.77	±0.85 bc	50.76	±5.12 c	7.00	±0.96 a	4.06	±0.41 a
HYTLO1/H_2_O	7.02	±1.00 ab	9.94	±1.45 ab	6.61	±0.36 a	4.56	±0.14 a
HYTLO1/T22	5.49	±0.66 a	7.22	±0.98 b	5.44	±0.63 ab	3.94	±0.34 a
HYTLO1/TH1	9.75	±0.84 c	8.04	±0.74 b	5.61	±0.31 a	4.79	±0.23 a
HYTLO1/GV41	3.95	±0.63 d	7.61	±0.84 b	4.16	±0.54 b	5.10	±0.23 b

**Table 7:** Effects of *Trichoderma* strains (TH1, M10, P1, T22, T53, HK5, CRN1, KV906 or GV41) or metabolites (HA, 6PP, HYTLO1), applied singly as seed coating, on the lentil mineral content. Plants were grown in field conditions and water treatments served as controls (CTRL). Data represent the mean value of three biological replicates (each one consisting of 10 plants) ± standard error (SE). Different letters in a column indicate statistically significant differences for *p* < 0.05. Significant increments compared with control (CTRL) are reported as %; nd: Not determined.

Treatment	Plant Dry Weight (g/Plant)		Seed Weight (g/Plant)		Mineral Content (ppm Solid)	
	Mean ± SE	%	Mean ± SE	%	Iron Mean ± SE (%)	Zinc Mean ± SE (%)
CTRL	16.9 ± 2.5 a	-	5.6 ± 0.5 a	-	83.1 ± 8.6 a	66.33 ± 3.7 a
TH1	26.6 ± 2.7 b	+57	15.2 ± 1.0 b	+169	82.2 ± 6.6 a	62.43 ± 1.8 a
M10	37.1 ± 0.4 c	+120	12.0 ± 0.4 c	+112	85.3 ± 10.8 a	66.38 ± 5.5 a
P1	nd	-	4.8 ± 0.3 a	-	73.6 ± 0.1 b	59.94 ± 1.0 b
T22	7.3 ± 0.2 e	-	5.8 ± 0.3 a	-	70.4 ± 3.6 b	56.40 ± 82.9 b
T53	13.3 ± 1.6 ad	-	9.7 ± 0.5 d	+71	87.3 ± 1.4 a	61.29 ± 2.7 a
HK5	16.5 ± 1.4 a	-	8.7 ± 2.4 de	+53	83.5 ± 2.0 a	68.82 ± 1.1 a
CRN1	17.5 ± 1.6 a	-	5.3 ± 2.1 a	-	82.1 ± 20.5 a	66.44 ± 7.6 a
KV906	20.4 ± 2.1 a	-	10.2 ± 0.7 cd	+81	98.4 ± 4.6 c (+11%)	73.97 ± 6.7 c (+18%)
GV41	17.9 ± 4.5 a	-	9.3 ± 1.9 cde	+65	76.1 ± 1.7 b	62.28 ± 4.2 a
HA	8.6 ± 0.6 e	-	5.1 ± 0.7 a	-	90.6 ± 0.1 a	66.72 ± 4.2 a
6PP	12.9 ± 0.7 d	-	7.6 ± 0.4 de	+36	73.6 ± 0.9 b	62.12 ± 0.1 a
HYTLO1	21.5 ± 3.4 ab	-	14.0 ± 2.9 b	+147	99.9 ± 2.0 c (+6%)	70.55 ± 2.2 c (+20%)
T22 + HA	16.3 ± 2.2 ad	-	6.6 ± 1.3 ae	-	88.6 ± 5.0 a	67.45 ± 3.3 a

## Data Availability

Not applicable.

## Supplementary Materials

The following are available online at https://www.mdpi.com/article/10.3390/microorganisms10010087/s1. Figure S1: Calibration curves of iron (Fe) and zinc (Zn) obtained by atomic absorption spectroscopy using commercial standard solutions; Table S1: Treatments performed in experiment #A with *Trichoderma* strains (T22, M10, P1) or metabolites (HA, 6PP); Table S2: Treatments performed in experiment #C with *Trichoderma* strains (T22, TH1, GV41) or metabolites (HA, 6PP, HYTLO1).
