# Supplementary material for: Mineral Biofortification and Growth Stimulation of Lentil Plants Inoculated with Trichoderma Strains and Metabolites
Source: Microorganisms. 2021 Dec 31;10(1):87. doi: 10.3390/microorganisms10010087 (PMC8779936; doi:10.3390/microorganisms10010087)
Supplement: Supplementary file 1 [file microorganisms-10-00087-s001.zip › microorganisms-1530294-supplementary.pdf]

Table S1. Treatments performed in experiment #A with *Trichoderma* strains (T22, M10, P1) or metabolites (HA, 6PP). 1X FeZn: solution of iron sulfate heptahydrate and zinc sulfate heptahydrate prepared as described in experimental design section.

No.	Seed treatment	1st soil watering	2nd soil watering
1	H_2O	H_2O	H_2O
2	T22	T22	H_2O
3	M10	M10	H_2O
4	P1	P1	H_2O
5	HA	HA	H_2O
6	6PP	6PP	H_2O
7	H_2O	H_2O	1X FeZn
8	T22	T22	1X FeZn
9	M10	M10	1X FeZn
10	P1	P1	1X FeZn
11	HA	HA	1X FeZn
12	6PP	6PP	1X FeZn

Table S2. Treatments performed in experiment #C with *Trichoderma* strains (T22, TH1, GV41) or metabolites (HA, 6PP, HYTLO1). 1X FeZn: solution of iron sulfate heptahydrate and zinc sulfate heptahydrate prepared as described in experimental design section.

No.	Seed treatment	1st soil watering	2nd soil watering
1	H_2O	H_2O	H_2O
2	H_2O	H_2O	1X FeZn
3	H_2O	T22	H_2O
4	H_2O	T22	1X FeZn
5	H_2O	TH1	H_2O
6	H_2O	TH1	1X FeZn
7	H_2O	GV41	H_2O
8	H_2O	GV41	1X FeZn
9	H_2O	HA	H_2O
10	H_2O	HA	1X FeZn
11	H_2O	6PP	H_2O
12	H_2O	6PP	1X FeZn
13	H_2O	HYTLO1	H_2O
14	H_2O	HYTLO1	1X FeZn
15	T22	H_2O	H_2O
16	T22	H_2O	1X FeZn
17	TH1	H_2O	H_2O
18	TH1	H_2O	1X FeZn
19	GV41	H_2O	H_2O
20	GV41	H_2O	1X FeZn
21	HA	H_2O	H_2O
22	HA	H_2O	1X FeZn
23	6PP	H_2O	H_2O
24	6PP	H_2O	1X FeZn

25	HYTLO1	H_2O	H_2O
26	HYTLO1	H_2O	1X FeZn
27	HA	T22	H_2O
28	HA	T22	1X FeZn
29	HA	TH1	H_2O
30	HA	TH1	1X FeZn
31	HA	GV41	H_2O
32	HA	GV41	1X FeZn
33	6PP	T22	H_2O
34	6PP	T22	1X FeZn
35	6PP	TH1	H_2O
36	6PP	TH1	1X FeZn
37	6PP	GV41	H_2O
38	6PP	GV41	1X FeZn
39	HYTLO1	T22	H_2O
40	HYTLO1	T22	1X FeZn
41	HYTLO1	TH1	H_2O
42	HYTLO1	TH1	1X FeZn
43	HYTLO1	GV41	H_2O
44	HYTLO1	GV41	1X FeZn

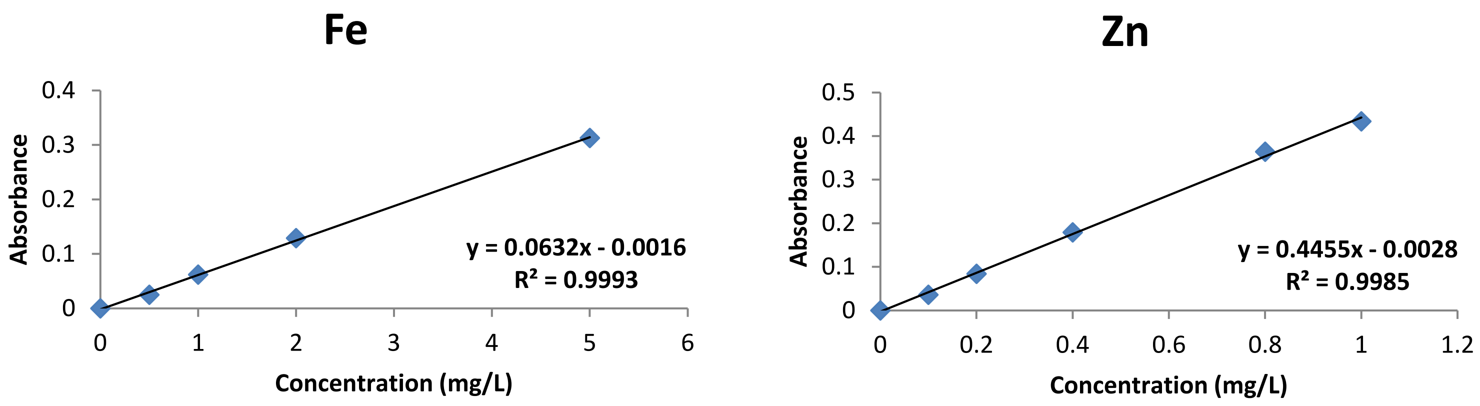

Figure S1. Calibration curves of iron (Fe) and zinc (Zn) obtained by Atomic Absorption Spectroscopy using commercial standard solutions.
